# Overexpression and Suppression of *Artemisia annua* 4-Hydroxy-3-Methylbut-2-enyl Diphosphate Reductase 1 Gene (*AaHDR1*) Differentially Regulate Artemisinin and Terpenoid Biosynthesis

**DOI:** 10.3389/fpls.2017.00077

**Published:** 2017-01-31

**Authors:** Dongming Ma, Gui Li, Yue Zhu, De-Yu Xie

**Affiliations:** Department of Plant and Microbial biology, North Carolina State University, Raleigh, NCUSA

**Keywords:** *Artemisia annua*, artemisinin, arteannuin B, 4-Hydroxy-3-methylbut-2-enyl diphosphate reductase, methylerythritol phosphate pathway, terpene

## Abstract

4-Hydroxy-3-methylbut-2-enyl diphosphate reductase (HDR) catalyzes the last step of the 2-C-methyl-D-erythritol 4- phosphate (MEP) pathway to synthesize isopentenyl pyrophosphate (IPP) and dimethylallyl diphosphate (DMAPP). To date, little is known regarding effects of an increase or a decrease of a *HDR* expression on terpenoid and other metabolite profiles in plants. In our study, an *Artemisia annua HDR* cDNA (namely *AaHDR1*) was cloned from leaves. Expression profiling showed that it was highly expressed in leaves, roots, stems, and flowers with different levels. Green florescence protein fusion and confocal microscope analyses showed that AaHDR1 was localized in chloroplasts. The overexpression of *AaHDR1* increased contents of artemisinin, arteannuin B and other sesquiterpenes, and multiple monoterpenes. By contrast, the suppression of *AaHDR1* by anti-sense led to opposite results. In addition, an untargeted metabolic profiling showed that the overexpression and suppression altered non-polar metabolite profiles. In conclusion, the overexpression and suppression of AaHDR1 protein level in plastids differentially affect artemisinin and other terpenoid biosynthesis, and alter non-polar metabolite profiles of *A. annua*. Particularly, its overexpression leading to the increase of artemisinin production is informative to future metabolic engineering of this antimalarial medicine.

## Introduction

**FIGURE 1:**
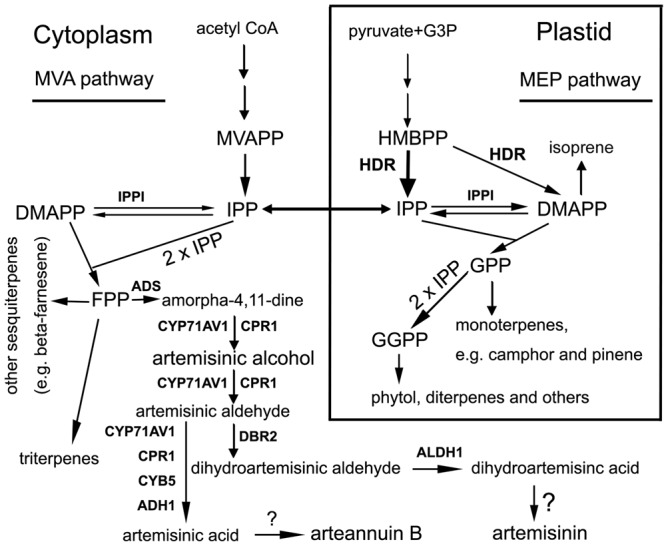
**A scheme showing pathways to artemisinin and other groups of terpenes starting with both the mevalonate and 2-C-methyl-D-erythritol 4-phosphate (MEP) pathways in *Artemisia annua*.** Metabolite abbreviations: G3P, glycerol-3-phosphate; HMBPP, 4-Hydroxy-3-methyl-but-2-enyl pyrophosphate; MVAPP, mevalonate pyrophosphate; IPP, isopentyl pyrophosphate; DMAPP, dimethylallyl pyrophosphate; GPP, geranyl pyrophosphate; FPP, farnesyl pyrophosphate; and GGPP, geranylgeranyl pyrophosphate. Enzyme abbreviations: HDR, 4-Hydroxy-3-methylbut-2-enyl pyrophosphate reductase. The wide arrow from HMBPP to IPP means HDR activity preferring to IPP; IPPI, isopentenyl pyrophosphate isomerase; ADS, armorpha-4, 11-diene synthase; ADH1, alcohol dehydrogenase 1; ALDH1, aldehyde dehydrogenase; CPR1, cytochrome P450 reductase 1; CYB5, cytochrome b5 mono-oxygenase, CYP71AV1, cytochrome P450 mono-oxygenase; DBR2, artemisinic aldehyde delta-11(13)-double bond reductase. Question marks (?) mean unknown.

4-Hydroxy-3-methylbut-2-enyl diphosphate (or pyrophosphate) reductase (HDR, EC 1.17.1.2) is a member of the NADP/NAD-dependent oxidoreductase family. It is also called isoprenoid synthesis H (IspH) or lysis-tolerant B (LytB) (Rohdich et al., 2002; Hsieh and Hsieh, 2015). It catalyzes the last step of the 2-C-methyl-D-erythritol 4-phosphate (MEP) pathway toward the formation of isopentenyl pyrophosphate (or diphosphate) (IPP or IDP) and dimethyl allyl pyrophosphate (diphosphate) (DMAPP) (**Figure 1**) (Guevara-Garcia et al., 2005). In the presence of NADPH, it catalyzes the reductive dihydroxylation of 4-Hydroxy-3-methyl-but-2-enyl pyrophosphate (HMBPP) to produce IPP and DMAPP, while in the presence of nicotinamide adenine dinucleotide phosphate (NADP^+^), it oxidizes dehydration of IPP to HMBPP. To date, HDR has been particularly characterized to catalyze HMBPP to both IPP and DMAPP, with a high proportion of IPP but a low proportion of DMAPP. In *Escherichia coli*, HDR was reported to reduce HMBPP to IPP and DMAPP with a ratio of 5:1-6:1 (Rohdich et al., 2002, 2003). A HDR homolog characterized from *Burkholderia glumae*, a Gram-negative rice-pathogenic bacterium, was shown to catalyze the formation of IPP and DMAPP with a ratio of 2.22:1–2.28:1 (Kwon et al., 2013). A few of HDR homologs have been characterized from plants. A HDR from tobacco BY-2 has been reported to convert HMBPP to IPP and DMAPP with a ratio of 5.7:1 (Tritsch et al., 2010). With a sodium dithionite (DT)-methyl viologen (MV) reducing buffer system, a recombinant *Ginkgo biloba* HDR homolog, namely GbIspH1 from chloroplast, was reported to catalyze the formation of IPP and DMAPP with a ratio of 15:1 (Shin et al., 2015). In comparison with the current understanding of biochemical characterization, fewer reports have showed effects of a *HDR* expression manipulation on plant metabolisms. A mutation of *HDR* in *Arabidopsis thaliana* was reported to lead to albino phenotype of plants and reduction of chlorophyll contents (Hsieh and Goodman, 2005). A virus-induced gene silencing of *HDR* in *Nicotiana benthamiana* was reported to lead to severe albino leaves, which were characterized by disorganization of palisade mesophyll, reduction of cuticle, decrease of plastids, and abnormal thylakoid membranes (Page et al., 2004). To our knowledge, effects of a high expression of *HDR* in chloroplast on plant metabolism remain open for investigation.

*Artemisia annua* L. is an effective antimalarial plant (Klayman, 1993; Alejos-Gonzalez et al., 2011). This medicinal plant biosynthesizes artemisinin, a sesquiterpene lactone with a unique endoperoxide bridge (Liu et al., 1979; Klayman, 1985; Xie et al., 2016). To date, artemisinin-based combination therapy (ACT) forms the first line treatment of malaria to save millions of people every year (WHO, 2006, 2013; Maude et al., 2009). In 2015, Professor Youyou Tu was awarded the Nobel Prize in Medicine for her discovery of artemisinin and innovation of antimalarial treatment (Molyneux and Ward, 2015; Xie, 2016). Artemisinin is mainly extracted from the aerial parts of *A. annua*. Given that the content of artemisinin is low (0–0.8% DW) in the leaves and flowers of wild-type *A. annua* in nature (Charles et al., 1990; Alejos-Gonzalez et al., 2011; Liu et al., 2011), global efforts have been undertaken to improve low production of artemisinin for ACT. These efforts include genetic breeding, understanding of biosynthesis, metabolic engineering, and synthetic biology coupled with semi-synthesis. All those efforts have led to considerable progresses. Breeding efforts have created hybrid varieties with a content higher than 1% (DW) in flower tissues during the blooming period of plants (Delabays et al., 1993; Wallaart et al., 1999; Graham et al., 2010). Promising metabolic engineering for more than 1% artemisinin (DW) was reported in *A. annua* (Tang et al., 2014). In addition, tobacco plant metabolic engineering showed another alternative to explore new strategies for artemisinin and its precursors (van Herpen et al., 2010; Farhi et al., 2011; Zhang et al., 2011). In comparison with plant metabolic engineering, the novel strategy using synthetic biology and semi-synthesis has recently achieved a fundamental industry goal to produce artemisinin. Since the first success of artemisinic acid engineering using recombinant laboratory yeast strains (Ro et al., 2006), continuous improvements of technology have led to production of tons of artemisinic acid from engineered industrial yeast strains (Paddon et al., 2013; Turconi et al., 2014). In 2014, Sanofi, Inc. developed its capacity to produce 60 tons synthetic semi-synthesis artemisinin (SSA) through synthetic biology of artemisinic acid (Turconi et al., 2014; Corsello and Garg, 2015; Peplow, 2016). Although, to date in reality, SSA is not cost-effective for ACT (Peplow, 2016), this success shows the potential application of synthetic biology for artemisinic acid.

To date, data from metabolic engineering, genetic breeding, and synthetic biology has fundamentally enhanced the understanding of artemisinin biosynthetic pathway (Bouwmeester et al., 1999; Zhang et al., 2011; Farhi et al., 2013; Paddon et al., 2013; Ma et al., 2015; Suberu et al., 2016). Its C_15_ skeleton results from two molecules of isopentenyl pyrophosphate (IPP) and one molecule of dimethyl allyl pyrophosphate (DMAPP), which can be synthesized from the mevalonic acid (MVA) and MEP pathways (**Figure 1**). Seven genes have been mainly cloned from glandular trichomes (Covello et al., 2007; Tang et al., 2014) and demonstrated to involve catalytic steps from beta-farnesyl pyrophosphate (FPP) to artemisinic acid and dihydroartemisinic acid (**Figure 1**). These genes encode armorpha-4, 11-diene synthase (ADS), alcohol dehydrogenase 1 (ADH1), aldehyde dehydrogenase (ALDH1), cytochrome P450 reductase 1 (CPR1), cytochrome b5 mono-oxygenase (CYB5), cytochrome P450 mono-oxygenase (CYP71AV1) and artemisinic aldehyde delta-11(13)-double bond reductase (DBR2) (**Figure 1**) (Suberu et al., 2016). Our recent pyrosequencing study using 6 different tissues annotated all of these seven genes, indicating that their expression is not limited to trichomes only (Ma et al., 2015). Furthermore, our pyrosequencing annotated two members of *CPR* (*CRP1* and *CPR2*), and multiple homologs of *ADH1* and *CYB5*, suggesting the biosynthetic pathway of artemisinin is more complicated than our present understanding.

Here, we report cloning of a *HDR* homology from *A. annua* and its effects on formation of artemisinin and other terpene molecules. We previously used pyrosequencing of self-pollinated *A. annua* to annotate two *HDR* homolog cDNAs, namely *AaHDR1* and *AaHDR2* (Ma et al., 2015). In this study, we cloned *AaHDR1* from leaves and then characterized that it encoded a protein localized in plastids. After its anti-sense and overexpression was introduced into *A. annua*, respectively, multiple transgenic plants were selected to show that these two types of transgene expressions differentially controlled contents of artemisinin and other terpene molecules.

## Results

### Isolation of *AaHDR1* cDNA, Sequence Analysis, and Phylogenetic Tree

**FIGURE 2:**
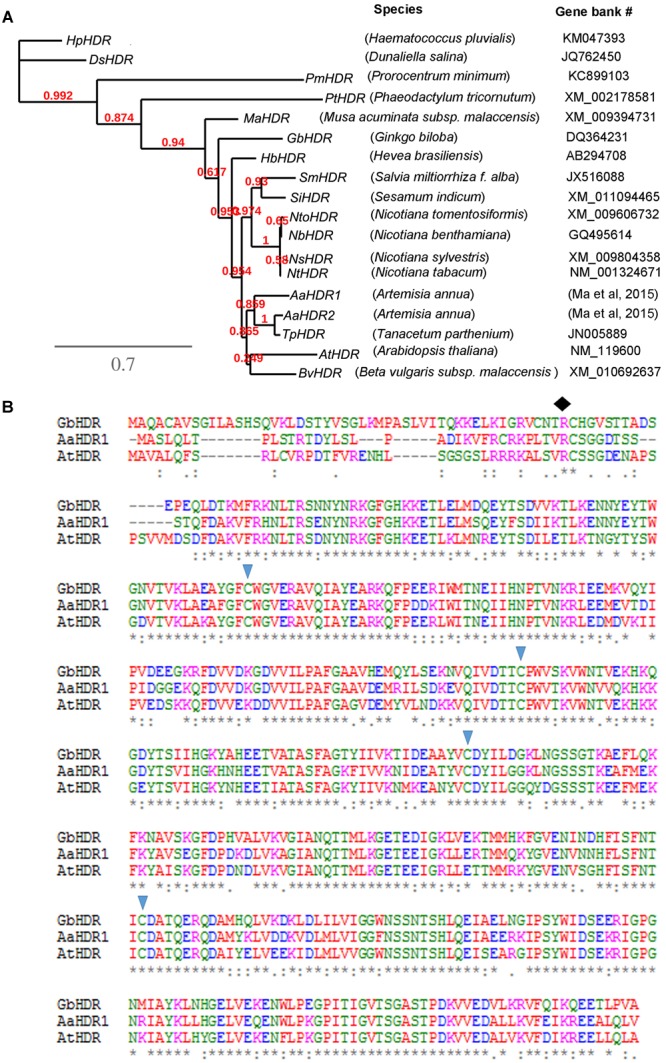
**Phylogenetic tree and amino acid alignment. (A)** A rooted phylogenetic tree was developed from nucleotide sequences of 18 4-Hydroxy-3-methylbut-2-enyl diphosphate reductase cDNA (*HDR*). **(B)** Alignment of the amino acids of AaHDR1 with two other HDRs from *Arabidopsis thaliana* (AtHDR) and *Ginkgo biloba* HDR (GbHDR). Four triangle shapes indicate conserved cysteine residues. The diamond shape indicates the possible site, from which the first 33 amino acids form a transit peptide.

Our previous pyrosequencing annotated two *HDR* homologs, *AaHDR1* and *AaHDR2*, the former of which contained a full length of nucleotide sequences (Ma et al., 2015). Based on our sequences, a cDNA fragment of *AaHDR1* consisting of 1664 bp nucleotides (GenBank #: KY288069) was cloned from leaf tissues of self-pollinated *A. ann*ua using RT-PCR. Sequence analysis showed that this fragment contained the open reading frame (ORF) consisting of 1365 bp nucleotides, which were deduced to encode 455 amino acids. An rooted phylogenetic tree was built using the nucleotide sequences of *AaHDR1* and *AaHDR2* from *A. annua* and 16 other homologs from four algae, one gymnosperm, and 13 angiosperms (one monocot species and 12 dicot species) (**Figure 2A**). This tree was characterized by four clades, four algae species, banana (monocot, *M. acuminata*), gingko (gymnosperm), and angiosperms. In angiosperms, *AaHDR1* and *AaHDR2* are clustered with the *Tanacetum parthenium HDR* homolog, the two plant species of which are in the family Asteraceae, suggesting a close phylogenetic relationship. In addition, an analysis of amino acid sequences using an online ProtParam tool^1^ revealed that its theoretical molecular weight and isoelectric point of the protein were 51405.46 Dalton and 5.63, respectively. Further sequence alignment with *A. thaliana* and *Ginkgo biloba* HDR homologs using the online TargetP ver. 1.0 software^2^ suggested that the 33 N-terminal amino acids (**Figure 2B**) likely form a chloroplast targeting signal peptide. This feature suggests that AaHDR1 is located in plastids. In addition, sequence alignment revealed that AaHDR1 contains four conserved cysteine residues at positons 111, 202,256, and 339 (marked with triangle shapes in **Figure 2B**), which have been well-characterized to be essential for the activity of HDR homologs (Grawert et al., 2004).

### Expression Profiles of cDNA and AaHDR1 Localization in Plastids

**FIGURE 3:**
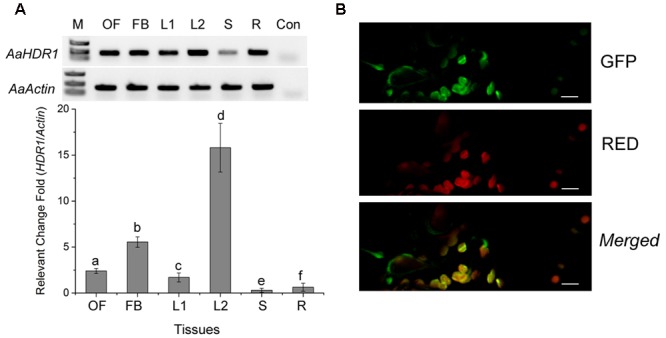
**Expression pattern of *AaHDR1* and subcellular localization. (A)** Semi-quantitative RT-PCR (upper panel) and real time quantitative RT-PCR (bottom panel) analysis of *AaHDR1* expression levels in different organs of *A. annua*, OF: full open flowers, FB: flower buds, L2: young leaves from plants grown on soil in the phytotron, L1, S and R: stems and roots of 8-week old seedlings grown on MS-medium contained in baby jars, Con: buffer control without the first strand of cDNA, a–f: different low case labeling indicating significant difference (*p*-value < 0.01). **(B)** Subcellular localization of AaHDR1-EGFP in the chloroplast. Green: the green fluorescence of GFP, RED: autofluorescence of chloroplast, Merged: the merged image of the green fluorescence of GFP and red autofluorescence of chloroplasts. Bar = 20 um.

Semi-quantitative RT-PCR analysis showed that *AaHDR1* is expressed in roots, stems and leaves of sterile seedlings grown on MS medium contained in baby jars, and open flowers, flower buds, and leaves of plants on soil in the phytotron (**Figure 3A**). Further real time qRT-PCR analysis showed different levels in these tissues (**Figure 3A**). This result was in agreement of our pyrosequencing results that *AaHDR1* was expressed in different positional leaves and flowers (Ma et al., 2015).

The ORF was fused to the N-end of an enhanced green florescent protein (EGFP) for a transient expression in *N. benthamiana*. Confocal microscopy analysis determined that the green fluorescence was detected in chloroplasts of leaf tissues, demonstrating the plastidial localization of AaHDR1 (**Figure 3B**).

### Overexpression of *AaHDR1* Increases Content of Artemisinin

**FIGURE 4:**
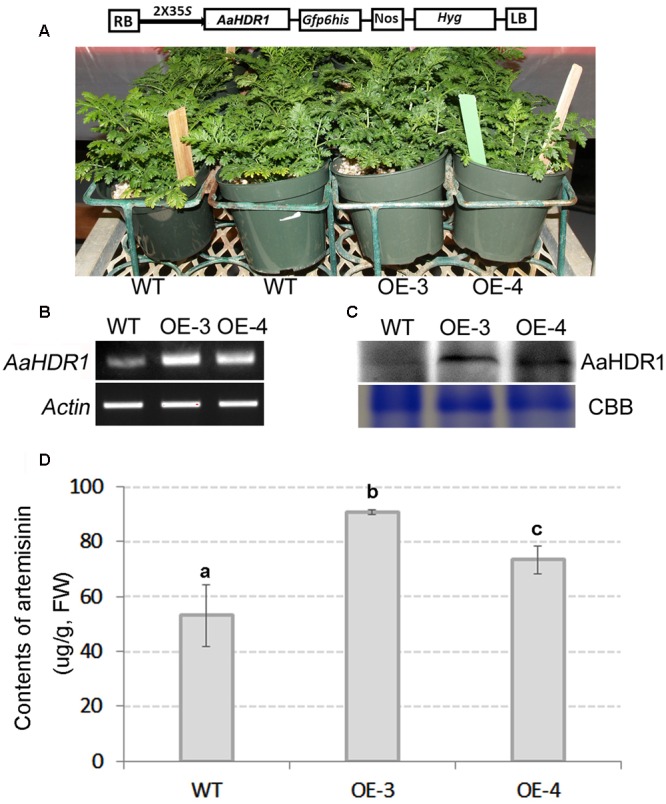
**Overexpression of *AaHDR1* leading to increase of artemisinin in leaves of transgenic plants. (A)** A cassette scheme shows overexpression of *AaHDR1* driven by 2 × 35S promoter in plasmid PMDC-AaHDR1 and phenotypes of transgenic lines vs. wild-type plants after grown on pot soil for 6 weeks; **(B)** RT-PCR images show higher expression levels of *AaHDR1* in young leaves of 6-week old transgenic lines than in wild-type plants; **(C)** western blot images show increase of protein level; **(D)** the artemisinin contents were increased in leaves of two transgenic lines. OE-3 and 4: two overexpression transgenic plants; WT, wild-type (a, b, and c labels indicate significant difference, *p* < 0.05).

The ORF of *AaHDR1* driven by a 2 × 35S promoter was transformed into self-pollinated *A. annua*. Multiple hygromycin-resistant plants were obtained from selection media containing 20 mg/L hygromycin. Two overexpression lines, namely OE-3 and OE-4, were selected for further analysis (**Figure 4A**). RT-PCR analysis demonstrated the elevated expression levels of *AaHDR1* in OE-3 and OE-4 transgenic plants (**Figure 4B**). Western blot demonstrated the increase of protein in these two transgenic lines (**Figure 4C**). After plants were grown in pot soil for 8 weeks in phytotron, leaves were collected for artemisinin and other metabolites analysis. HPLC–MS analysis showed the significant increase of artemisinin content in leaves of these two lines than in those of wild-type plants (**Figure 4D**).

### Overexpression of *AaHDR1* Alters Non-polar Metabolite Profile, Increases Levels of Arteannuin B and Eight Other Terpenes, and Reduces Level of Caryophyllene

**FIGURE 5:**
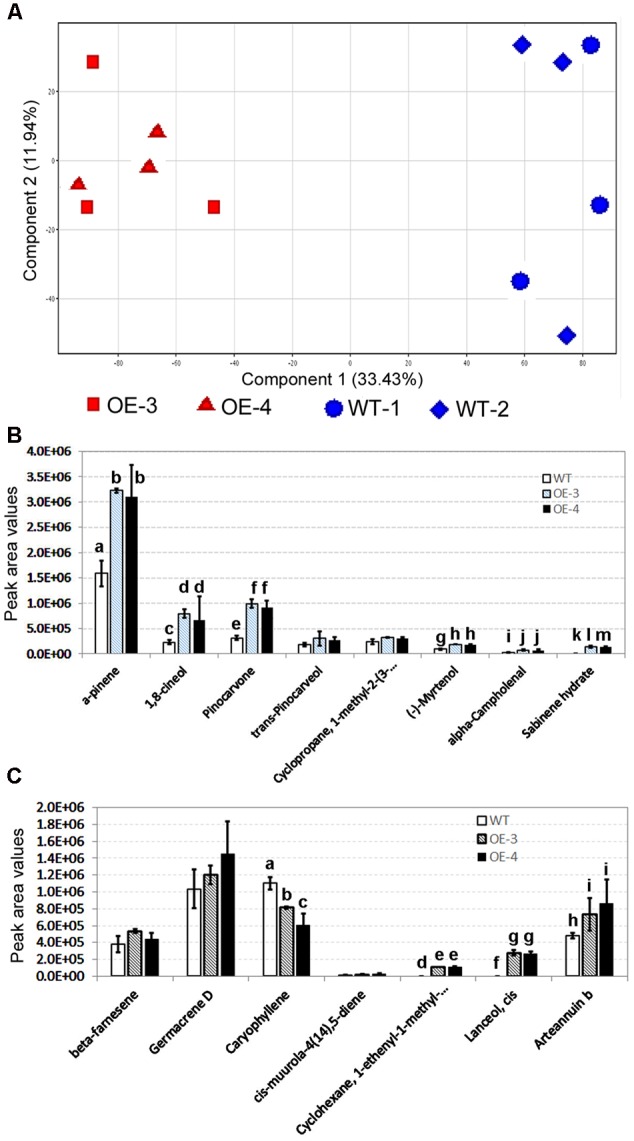
**Effects of the overexpression of *AaHDR1* on non-polar metabolite and terpene profiles in leaf samples.** Non-polar metabolites were extracted from mixed samples of leaf #7 -13. **(A)** A PCA plot shows non-polar metabolite profile differentiation between samples of transgenic and WT plants; **(B,C)** effects of the *AaHDR1* overexpression on levels of eight monoterpenes **(B)** and seven sesquiterpenes **(C)**. Bars, which are labeled with different low case letters for each metabolite (a, b, c, d, or e) in **B, C**, mean significant difference (*p*-values < 0.05), while, bars, which are labeled with the same low case letters, mean insignificant difference. OE-3 and -4, two overexpression transgenic plants; WT, wild-type.

Untargeted GC–MS analysis annotated 85 non-polar metabolites from leaves of two transgenic and two wild-type control plants, after grown on pot for 8 weeks. These compounds and their peak values were used as data matrix for principal component analysis (PCA). The resulting two-dimensional PCA plot consisting of PC1 (33.43%) and PC2 (11.94%) showed a distinct ordinate separation between two transgenic lines and two wild-type plants in the PC1 (**Figure 5A**), indicating alternations of non-polar metabolite profiles by the overexpression of *AaHDR1*.

Of 85 non-polar metabolites detected, 15 were terpenoid molecules including eight monoterpenes and seven sesquiterpenes. The peak value of each metabolite was used to compare their levels in leaves of transgenic vs. wild-type plants. The resulting data showed that the levels of six monoterpenes were significantly higher in transgenic leaves than in wild-type ones (**Figure 5B**). The levels of two other monoterpenes were only slightly higher in transgenic leaves. Of seven sesquiterpenes, one is arteannuin b, the peak values of which were 1.5–1.8 folds (*p*-value < 0.05) higher in transgenic leaves than in wild-type ones (**Figure 5C** and **Supplementary Figure S1**). In addition, transgenic leaves produced significantly higher levels of *cis*-lanceol and 1-enthyl-1-methyl-cyclohexane than wild-type control. The levels of beta-farnesene and germacrene D were lightly increased in transgenic leaves. In contrast with these metabolites, the level of caryophyllene was significantly decreased in transgenic leaves.

### Anti-sense of *AaHDR1* Decreases Content of Artemisinin

**FIGURE 6:**
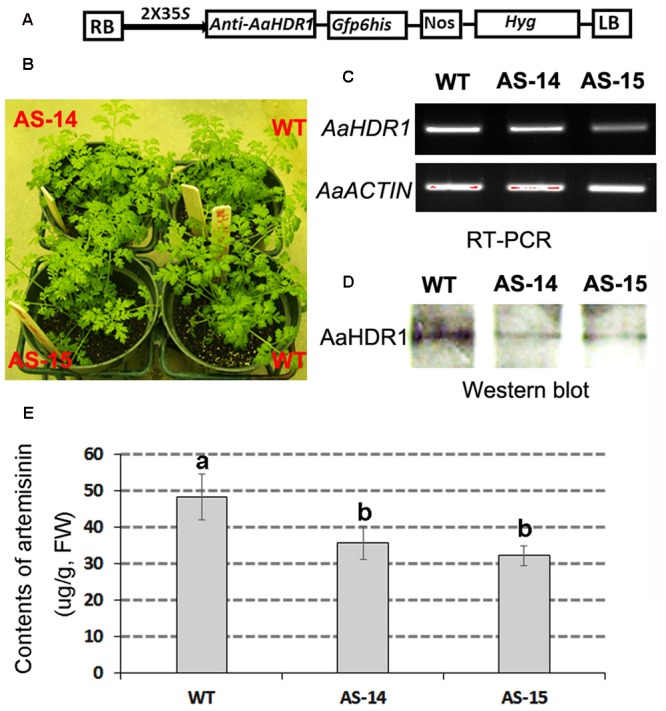
**Anti-sense of *AaHDR1* leading to decrease of artemisinin in leaves of transgenic plants. (A)** a cassette scheme shows anti-sense orientation in plasmid PMDC-antiAaHDR1 to down-regulate *AaHDR1* expression; **(B)** phenotypes of two transgenic lines vs. wild-type plants after grown on pot soil for 6 weeks; **(C)** RT-PCR images show reduced expression levels of *AaHDR1* in young leaves of 6-week old transgenic plants; **(D)** western blot images show decrease of protein level; **(E)** contents of artemisinin is reduced in two transgenic lines. Bars, which are labeled with different low case letters for each metabolite, mean significant difference (*p*-values < 0.05), while, bars, which are labeled with the same low case letters, mean insignificant difference. AS-14 and 15, two anti-sense transgenic plants; WT, wild-type.

An anti-sense binary vector of *AaHDR1* (**Figure 6A**) was constructed and introduced into *A. annua.* Multiple transgenic lines were obtained, two of which, namely AS-14 and AS-15 (**Figure 6B**), were selected for further analysis. RT-PCR analysis showed the decreased expression levels of *AaHDR1* in leaves (**Figure 6C**). Western blot analysis determined that the level of AaHDR1 was reduced in transgenic leaves than in wild-type ones (**Figure 6D**). After plants were grown on pot soil for 9 weeks (**Supplementary Figure S3**) in the phytotron, leaves were collected for artemisinin and other metabolites analysis. HPLC–MS analysis demonstrated that contents of artemisinin were decreased 27–33% in transgenic leaves (**Figure 6E**).

### Antisense of *AaHDR1* Alters Non-polar Metabolite Profile and Decreases Levels of Arteannuin B and Seventeen Other Terpenes

**FIGURE 7:**
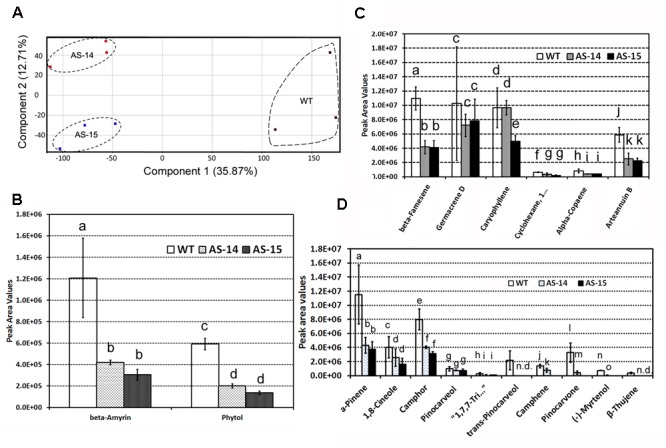
**Effects of the down-regulation of *AaHDR1* on non-polar metabolite and terpene profiles in leaf samples of transgenic plants.** Non-polar metabolites were extracted from mixed samples of leaf #7 -13. **(A)** A PCA plot shows non-polar metabolite profile differentiation between samples of transgenic and WT plants; **(B–D)** effects of antisense on β-amyrin and phytol **(B)**, six sesquiterpenes **(C)**, and 10 monoterpenes **(D)** in leaves of two transgenic lines. Bars, which are labeled with different low case letters for each metabolite (a, b, c, d, or e) in **B–D** mean significant difference (*p*-values < 0.05), while, bars, which are labeled with the same low case letters, mean insignificant difference. AS-14 and 15, two anti-sense transgenic plants; WT, wild-type.

Untargeted GC–MS analysis annotated 82 non-polar metabolites. These metabolites were used as data matrix for PCA. The resulting two dimensional plot including PC1 (35.87) and PC2 (12.71%) showed an ordinate distinction of metabolite profiles in the PC1 (**Figure 7A**).

Of 82 metabolites, there were 18 terpenes, including β-amyrin and phytol (**Figure 7B**), six sesquiterpenes (**Figure 7C**), and 10 monoterpenes (**Figure 7D**). Peak values of these metabolites were recorded to compare their levels in transgenic vs. wild-type leaves. In comparison, the level of arteannuin B (**Figure 7C** and **Supplementary Figure S2**) was reduced approximately twofold in transgenic leaves. In addition, the levels of five other sesquiterpenes were significantly or slightly decreased in transgenic leaves. Furthermore, the levels of 10 monoterpenes were significantly reduced in transgenic leaves (**Figure 7D**). The levels of β-amyrin and phytol were significantly reduced in transgenic leaves (**Figure 7B**).

## Discussion

4-Hydroxy-3-methylbut-2-enyl diphosphate reductase catalyzes the last step of the MEP pathway from HMBPP to IPP and DMAPP in organisms (**Figure 1**) (Wolff et al., 2003; Seemann et al., 2009; Vranova et al., 2013; Banerjee and Sharkey, 2014). However, to date, there is no evidence regarding to what an extent HDR controls the contents of artemisinin and other terpenoid molecules in *A. annua*. Our experiments undertaken in present study show that the expression level of AaHDR1 is closely associated with the contents of artemisinin and numerous other terpenes. The overexpression of *AaHDR1* increased the total content of protein (**Figure 4C**), while the suppression by its anti-sense decreased the total content of the enzyme (**Figure 6D**). Corresponding to these two consequences, the contents of artemisinin (**Figure 4D**), arteannuin B and three other sesquiterpenes (**Figure 5C**), and six monoterpenes (**Figure 5B**) were increased in the *AaHDR1* overexpressing plants. By contrast, the contents of artemisinin (**Figure 6E**), arteannuin B and four other sesquiterpenes, 10 monoterpenes, phytol, and beta-amyrin (**Figures 7B–D**) were decreased in the *AaHDR1* suppressing (anti-sense) plants. These transgenic data indicate that the *in planta* expression level of AaHDR1 affects the production of artemisinin and other terpenes. In addition to *A. annua*, genetic and transgenic studies in other plants have shown that a reduction of HDR level can cause chlorophyll pigmentation alterations. A mutation of *HDR* in *A. thaliana* was reported to lead to severe albino phenotypes (Hsieh and Goodman, 2005). Gene silencing of *HDR* in tobacco was also reported to lead to albino phenotypes of transgenic plants (Page et al., 2004). Those albino phenotypes in both *A. thaliana* and tobacco plants resulted from the reduced biosynthesis of chlorophyll from the MEP pathway in mutant or gene silencing plants. Taken together, it is apparent that the activity of HDR in the chloroplasts is critical in the control of profiles of downstream metabolites (**Figure 1**), such as monoterpenes, sesquiterpenes, chlorophyll, and others.

The biosynthetic differentiation of artemisinin and other sesquiterpenes oppositely regulated by the overexpression and suppression of *AaHDR1* provides transgenic evidence to support the observation that IPP can be exchanged from plastids to the cytosol (Wanke et al., 2001; Nagata et al., 2002; Bick and Lange, 2003; Laule et al., 2003). HDR catalyzes HMBPP to IPP and DMAPP (**Figure 1**), two essential molecules for all life (Vranova et al., 2013). In plants, these molecules are synthesized by the aid of the cytosolic MVA and independently by the plastidial MEP pathway (**Figure 1**) (Vranova et al., 2013). Our EGFP fusion and confocal microscope analysis showed that AaHDR1 was localized in the chloroplasts. This result indicated that the *in vivo* catalysis from HMBPP to IPP and DMAPP by AaHDR1 was localized in the chloroplasts. As well-characterized, the backbone structure of sesquiterpenes is derived from one DMAPP and two IPP molecules (**Figure 1**). Accordingly, it can be suggested that the increase of artemisinin and other sesquiterpenes by the overexpression of *AaHDR1* in transgenic plants OE-3 and OE-4 would result from an enhanced pool size of IPP and DMAPP, which were transported from the chloroplasts. The transportation of IPP and geranyl pyrophosphate (GPP) from the chloroplasts to the cytosol was metabolically observed in other studies. When *A. annua* was fed with ^13^CO_2_ and artemisinin was then extracted for nuclear magnetic resonance spectrum analysis, the resulting isotopologue patterns showed that its precursor, farnesyl diphosphate, was predominately derived from the assimilation of ^13^CO_2_ (Schramek et al., 2010). GPP is generally considered being synthesized toward the biosynthesis of monoterpenes in the plastids (**Figure 1**). In transgenic tomato fruits, when the content of GPP was increased in the plastids, the cytosol was observed to produce monoterpenes, indicating the export of GPP from plastids to the cytosol (Gutensohn et al., 2013). In addition, we recently introduced a synthetic plant-insect GPPS into Camelina. The overexpression of the synthetic GPPS led to significant reduction of isoprene (a direct product of DMAPP) (**Figure 1**) but significant increase of amyrin in tissues, implying that more IPP molecules were exported to the cytosol from plastids (Xi et al., 2016).

In summary, A *HDR* homolog, namely *AaHDR1*, is cloned from self-pollinated *A. annua*. The enzyme is localized in plastids. Both overexpression and suppression in *A. annua* demonstrate that its transcription and protein levels are closely associated with contents of artemisinin, arteannuin B and other sesquiterpenes, and numerous monoterpenes. All results suggest that the levels of IPP and DMAPP from the AaHDR1 catalysis are critical for metabolic engineering of high artemisinin production.

## Materials and Methods

### Plant Materials and Growth Conditions

A self-pollinated *A. annua* variety was bred from an ecotype collected from USA (Alejos-Gonzalez et al., 2011). Progenies of plants have been grown in the phytotron for seeds as described previously (Alejos-Gonzalez et al., 2011). Wild-type seedlings of the F3 progeny grown on agar-solidified MS medium contained in baby jars in a growth chamber as described previously (Alejos-Gonzalez et al., 2013) were used for genetic transformation and gene expression profiling. Wild-type and transgenic plants grown on soil in the phytotron were used to analyze transgene expression, western blot, artemisinin analysis, and metabolic profiling. The photoperiod in the growth chamber and phytotron was 16/8 h (light/dark). The light intensities in the growth chamber and the phytotron were 50 and 200 μmol/m^-2^ s^-1^, respectively.

### Isolation of *AaHDR1* cDNA

We recently reported two *HDR* contigs, *AaHDR1* and *AaHDR2*, assembled from six EST libraries (including leaves and flowers) of the self-pollinated *A. annua* (Ma et al., 2015). The first strand cDNA libraries remained after sequencing were stored in -80°C freezer and then used to clone genes. Based on EST sequence, we designed a pair of primers: HDR-F (5′-ATG GCG TCT TTG CAG CTA ACA-3′) and HDR-R (5′-CTA CAC CAA TTG CAG GGC CTC-3′). By following our gene cloning protocol reported previously (Ma et al., 2015), RT-PCR was carried out to clone the ORF sequence of *AaHDR1*.

### Analysis of Sequence, Phylogeny, and Sequence Alignment

Deduced amino acid sequences were analyzed using an online ProtParam tool^1^ to predict molecular properties. Amino acid sequences were also analyzed using an online TargetP ver. 1.0 software^2^ to predict conserved active amino acid residues. The full length nucleotide sequence (1664 pb) of *AaHDR1* was used for blastn search at NCBI. The blasting result included 16 homologous sequences with an e-value < 10E-8. All sequences were converted to fasta format for phylogenetic analysis using the online phylogeny.fr program^3^. In addition, amino acids for these homologs were released from NCBI and also converted to fasta format for sequence alignment using an online Cluster Omega program^4^.

### Subcellular Localization Analysis

A GATEWAY cloning technique was used to clone the *AaHDR1* ORF into pENTY/D-TOPO vector (Gateway, Invitrogen, USA) by following the manufacturer’s protocol. The resulting recombinant plasmid was named pENTY/D-TOPO-HDR. Then, the ORF of *AaHDR1* in the pENTY/D-TOPO-HDR vector was subsequently cloned to the destination vector pSITEII-N1-EGFP (with EGFP epitope tagging in the C-terminus) by LR reactions by following the manufacturer’s protocol. The resulting plasmid was named as pSITEII-N1-HDR/EGFP, in which the stop codon of *AaHDR1* was removed and ligated to the adjacent place immediately prior to the start codon ATG of *EGFP*. The new plasmid was then introduced to *Agrobacterium tumefaciens* strain GV3101. A positive colony was obtained using 100 mg/L streptomycin for selection. The positive colony was activated for agroinfiltration of *N. benthamiana* leaves to analyze transient protein expression by following the protocol reported previously (Martin et al., 2009). After 30 h of infection, leaf tissues were analyzed using a confocal microscope (Carl Zeiss). The GFP fluorescence was excited at 488 nm and observed between 495 and 550 nm as reported (Wang et al., 2015).

### Development of Binary Vectors and Genetic Transformation of *A. annua*

The ORF of *AaHDR1* with its terminal codon was cloned to the pENTY/D-TOPO vector (Gateway, Invitrogen) to obtain a recombinant pENTY-AaHDR1 plasmid, which was then introduced to competent cells of *E. coli* DH5α. Given that pENTY/D-TOPO vector can allow both sense and anti-sense orientation ligation, 10 positive individual colonies were selected to isolate plasmids for sequencing to identify sense or anti-sense insertion orientations of the *AaHDR1* ORF. Sequencing showed that eight colonies contained a sense orientation of the ORF cDNA, namely pENTR-AaHDR1 and two colonies contained an anti-sense orientation the ORF cDNA, namely pENTR-antiAaHDR1. The destination vector used for development of our binary vectors was PMDC-84 (Curtis and Grossniklaus, 2003). The pENTR-AaHDR1 and pENTR-antiAaHDR1 and the destination vector PMDC-84 were digested by LR Clonase II enzyme mix (Invitrogen) by following the manufacturer’s protocol. This reaction generated recombinant binary vectors, PMDC-AaHDR1 (**Figure 4A**) (from PMDC-84 and pENTR-AaHDR1) and PMDC-antiAaHDR1 (from PMDC-84 and pENTR-antiAaHDR1) (**Figure 6A**), in which the sense and antisense orientations of *AaHDR1* ORF were driven by a 2 × 35S promoter.

The overexpression and anti-sense constructs, PMDC-AaHDR1 (**Figure 4A**) and PMDC-antiAaHDR1 (**Figure 6A**), were introduced to *A. tumefaciens* strain LBA4404 for genetic transformation of *A. annua* as described previously (Xie et al., 2004; Ma et al., 2015). In summary, seeds were sterilized and germinated on an agar-solidified basal MS medium (Murashige and Skoog, 1962) and leaves at the 5, 6, and 7 nodes of one and a half month old seedlings were used as explants for infection of *Agrobacterium*. The infected leaf disks were transferred onto an agar-solidified selection medium, which was composed of basal MS medium, 1.0 mg/L 6-benzylaminopurine (BAP), 0.05 mg/L naphthalene-1-acetic acid (NAA), 20 mg/L hygromycin, and 200 mg/L timentin. Survival infected leaf disks were transferred to freshly prepared selection medium every 10 days of interval (subculture). After approximately 50 days of selection, multiple hygromycin-resistant adventitious shoots were obtained, excised from calli, and then inoculated on a rooting medium (half-strength MS, 0.5 mg/L NAA, 20 mg/L hygromycin, and 200 mg/L timentin). Hygromycin-resistant adventitious shoots started to grow roots to develop into plantlets after 1–2 weeks of induction. Regenerated plantlets were planted on pot soil and grown in the phytotron by following our growth protocol as described above. The transgenic plant growth in the phytotron was observed carefully to compare their development. Those transgenic plants that grew at similar developmental stage to wild-type plants (**Figures 4A** and **6B**) were used for transgene expression, western blot, and metabolite analysis described below.

### Sampling for RT-PCR, GC–MS, and LC–MS Analyses

For *AaHDR1* expression profile analysis in wild-type plants, 8-week old seedlings grown in baby jars were used to collect roots, leaves, and stems, while 10-week old plants grown in the phytotron were used to collect open flowers, flower buds, and leaves. Three biological replicates were collected for each tissue. For transgene expression and western blot analysis, young leaves were collected from 6-week old transgenic vs. wild-type plants grown on pot soil in the phytotron. For GC–MS, and LC-MS analysis, leaves were collected 8-week old overexpression lines and 9-week old anti-sense expression lines (**Supplementary Figure S3**). For each plant, leaves were collected from nodes #7-13 of and then pooled together. All samples were frozen in liquid nitrogen and stored in 80°C freezer until use.

### Semi-quantitative and Real Time Quantitative RT-PCR

Total RNA was isolated from 100 mg of roots, leaves and stems of seedlings grown in baby jars, and open flowers, flower buds, and leaves of plants grown in the phytotron with the RNeasy Mini Kit (Qiagen, Germantown, MD, USA) in accordance with the manufacturer’s instructions. Total RNA was then treated with DNase I (Roche, CA, USA) to remove potential genomic DNA. First-strand cDNA synthesis was carried out with 1.0 μg of total RNA Super SMART PCR cDNA synthesis kit (Clontech, Mountain View, CA, USA) according to the manufacturer’s protocol. The resultant first-strand cDNA was used as the template for PCR.

A pair of gene specific primers was designed from the 3′-untranslated region of *AaHDR1* cDNA, namely AaHDR-Fx (5′-GGCATGTACCTGGCAAGAGAG-3′) and AaHDR-Rx (5′-GTCGTTTATAGCAACCAGAGCC-3′). In addition, the house keeping *ACTIN* gene was used as reference control and was amplified using a pair of primers: actin F (5′-AACTGGGATGACATGGAGAAGATAT-3′) and actin R (5′-TCACACTTCATGATGGAGTTGTAGG-3′). These two pairs of primers were used for both semi-quantitative and real time quantitative RT-PCR. For semi-quantitative PCR, the thermal program was composed of 5 min at 95°C, 30 cycles at 94°C for 30 s, 55°C for 40 s and 72°C for 110 s, and a 10 min extension at 72°C. PCR products were electrophoresed on a 1% agarose gel and visualized after ethidium bromide staining. Images of gels were photographed using a GelDoc EQ imager (Bio-Rad). This experiment was repeated three times. For quantitative analysis, real time qPCR was performed on a Power SYBR Green PCR Master Mix (Applied Biosystems) according to the manufacturer’s guidelines. The thermal cycles were composed of 50°C for 2 min and 95°C for 10 min, followed by 95°C for 15 s and 60°C for 1 min with 45 cycles, and then finished with 40°C. Five replicates and three replicates were performed for *AaHDR1* and *ACTIN*, respectively. *ACTIN* was used internal standard for normalization. The relative expression of *AaHDR1* was calculated using the ddCt algorithm.

### Western Blot Analysis

Polypeptides consisting of CIDGGEKQFDVVDKG were designed for preparing polyclonal antibody. This peptide was synthesized and then used to develop polyclonal antibody at the GenScript Company (Piscataway, NJ 08854, USA). Total protein extraction from leaves and immunoblot analysis were performed by following a method previously reported for deoxy-D-xylulose-5-phosphate synthase analysis (Estevez et al., 2001). In brief, total protein was extracted from the leaves of *A. annua*, separated by SDS-PAGE, transferred to nitrocellulose membrane, and probed with rabbit anti-AaHDR1 antibodies. An anti-rabbit IgG HPR conjugate was used as a second antibody (Promega, USA). The enhanced chemiluminescence (ECL) system was used for detection in the immunoblot analysis (Thermos Scientific, Rockford, IL, USA).

### Extraction of Metabolites and GC–MS Analysis

We developed a protocol to extract and profile non-polar metabolites from tissues of *A. annua* (Ma et al., 2015). In brief, hexane was used to extract non-polar metabolites from mixed samples of leave on nodes #7-13. Three replicates were prepared for each transgenic line (anti-sense and overexpression) and wild-type plants. GC–MS was performed using a gas chromatograph 6890 coupled with 5975C MSD (Agilent Technologies, USA). A RTX-5 capillary column (30 m × 0.25 mm × 0.25 μm) was used to separate metabolites. The splitless mode was used in the inlet. The injection temperature was set at 250°C. The temperature was initially set at 60°C and then ramped to 260°C at a constant rate of 10°C/min, and held at 260°C for 25 min. Pure helium was used as the carrier gas, with a flow rate of 1 ml/min. A positive electron impact ion source (70 EV) was used to ionize compounds, and mass fragments were scanned in the range of 40–800 (m/z), with 4 min of solvent delay.

### Deconvolution of Metabolite Peaks and Statistical Analysis

We have developed a method to use an Agilent MassHunter Mass Profiler (MHMP) and Mass Profiler Professional (MPP) software to deconvolute peaks detected by GC–MS (Ma et al., 2015). This software has been used for characterizing metabolic profiles (Chen et al., 2012; Chan et al., 2013; Hu et al., 2013; Bannur et al., 2014). The fold-change for all metabolites was obtained using log 2 values, which were calculated values that resulted from the transformation of original chromatographic peak accounts compared with each metabolite median value. The median value of each metabolite was obtained from four biological samples analyzed. The peak value of each metabolite was compared with the median value for log 2 normalization. Then, normalized data were used for PCA.

### HPLC–MS Analysis of Artemisinin

We have developed a HPLC–MS protocol for artemisinin analysis on a 2010 eV LC/UV/ESI/MS instrument (Shimadzu) (Alejos-Gonzalez et al., 2011, 2013). Mixed samples of leaves on nodes #7-13 from transgenic vs. wild-type plants were stored in freezer described above and then used to extract and estimate artemisinin contents. Three replicates were carried out for each transgenic line and wild-type plants.

## Author Contributions

D-YX developed the entire project and experimental plans, provided technical training, participated in data analysis, and drafted and finalized this manuscript. DM performed most of experiments, analyzed data and drafted this manuscript. GL performed RNA isolation, semi-quantitative PCR, real time quantitative PCR, and preparation of figures. YZ performed RNA isolation, real-time quantitative PCR, and preparation of figures.

## Conflict of Interest Statement

The authors declare that the research was conducted in the absence of any commercial or financial relationships that could be construed as a potential conflict of interest.

## Footnotes

1 http://cn.expasy.org/tools/protparam.html

2 http://www.cb.dtu./dk/services/TargetP

3 http://www.phylogeny.fr

4 http://www.ebi.ac.uk/Tools/msa/clustalo/

## Supplementary Material

FIGURE S1 **Total ion chromatographs show arteannuin b and its level increase in *AaHDR1* overexpression transgenic plants compared to wild-type control plants**.

FIGURE S2 **Total ion chromatographs show arteannuin b and its level decrease in *AaHDR1* anti-sense transgenic plants compared to wild-type control plants**.

FIGURE S3 **Anti-sense transgenic vs. wild-type plants grown in the phytotron after 9 weeks of transferring from baby jar to pot soil.** These plants were used for leaf samples for metabolite extraction and metabolic profiling.
