# Supplementary material for: Overexpression and Suppression of Artemisia annua 4-Hydroxy-3-Methylbut-2-enyl Diphosphate Reductase 1 Gene (AaHDR1) Differentially Regulate Artemisinin and Terpenoid Biosynthesis
Source: Front Plant Sci. 2017 Jan 31;8:77. doi: 10.3389/fpls.2017.00077 (PMC5281613; doi:10.3389/fpls.2017.00077)
Supplement: FIGURE S1 — Total ion chromatographs show arteannuin b and its level increase in AaHDR1 overexpression transgenic plants compared to wild-type control plants. [file Data_Sheet_1.pdf]

Supplementary Figures

Overexpression and Suppression of *Artemisia annua* 4-Hydroxy-3-Methylbut-2-enyl Diphosphate Reductase 1 Gene (*AaHDR1*) Differentially Regulate Artemisinin and Terpenoid Biosynthesis

Dongming Ma, Gui Li, Yue Zhu, and De-Yu Xie

Department of Plant and Microbial biology, North Carolina State University, USA

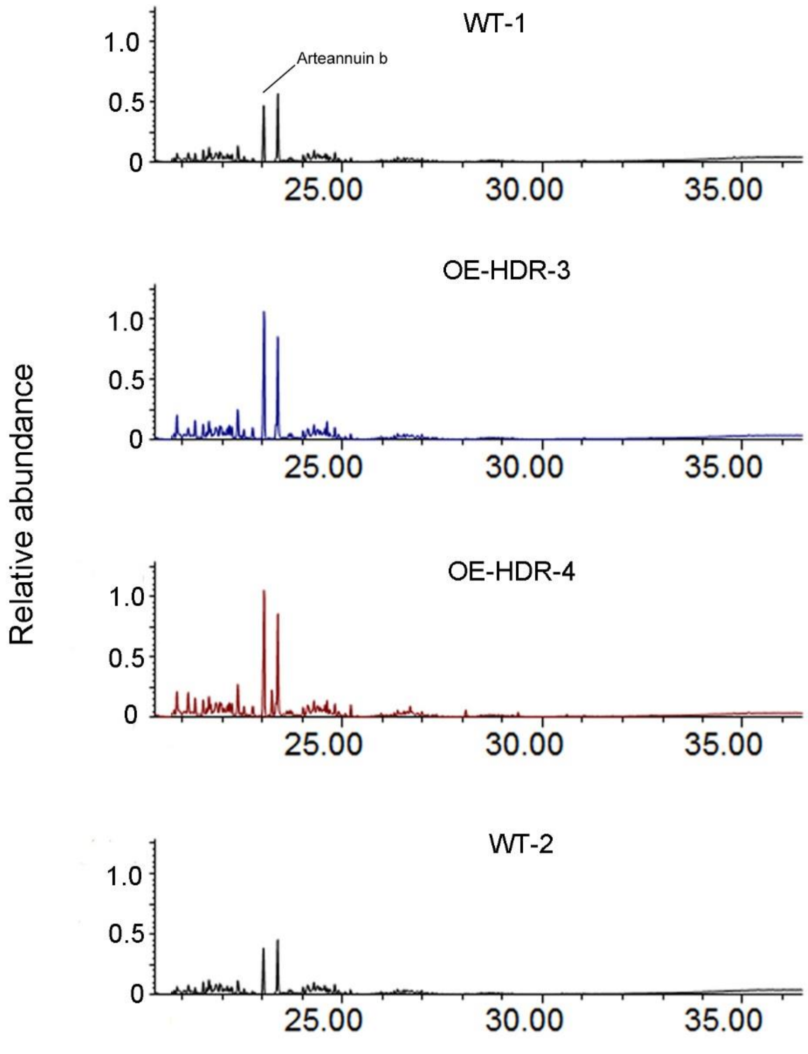

Supplementary Figure S1 Total ion chromatographs show arteannuin b and its level increase in *AaHDR1* overexpression transgenic plants compared to wild-type control plants.

PCR, western blot, and metabolite extraction.

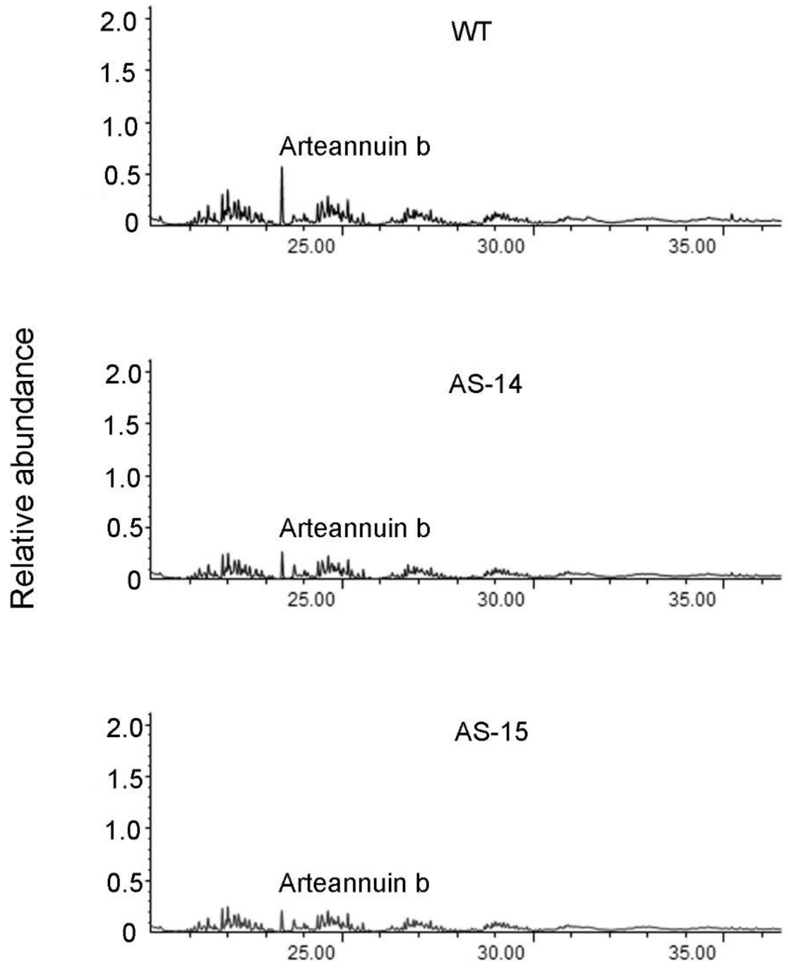

Supplementary Figure S2

Total ion chromatographs show arteannuin b and its level increase in *AaHDR1* anti-sense transgenic plants compared to wild-type control plants.

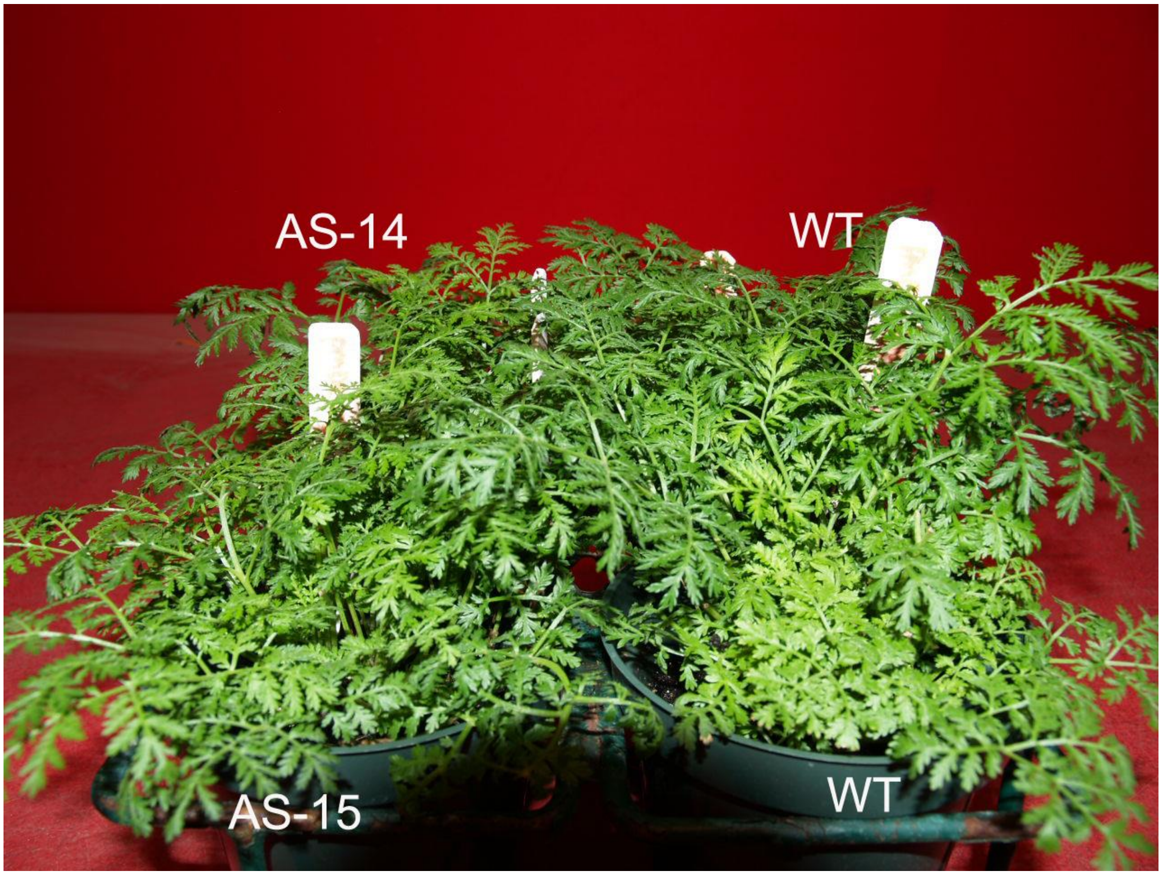

Supplementary Figure S3 Anti-sense transgenic vs. wild-type plants grown in the phytotron after 9 weeks of transferring from baby jar to pot soil. These plants were used for leaf samples for metabolite extraction and metabolic profiling.
